# An unusual presentation in relapse case of histoid leprosy

**DOI:** 10.1093/skinhd/vzaf067

**Published:** 2025-10-28

**Authors:** Mahesh Mathur, Sumit Paudel, Nabita Bhattarai, Sandhya Regmi, Sambidha Karki, Sharad Shrestha

**Affiliations:** Department of Dermatology, College of Medical Sciences, Bharatpur, Nepal; Department of Dermatology, College of Medical Sciences, Bharatpur, Nepal; Department of Dermatology, College of Medical Sciences, Bharatpur, Nepal; Department of Dermatology, College of Medical Sciences, Bharatpur, Nepal; Department of Dermatology, College of Medical Sciences, Bharatpur, Nepal; Department of Dermatology, College of Medical Sciences, Bharatpur, Nepal

## Abstract

Histoid leprosy (HL) is a very rare and highly infectious variant of lepromatous leprosy, presenting as skin-coloured, succulent nodules and plaques on apparently healthy skin. It is histologically characterized by a dense bundle of histiocytes arranged in storiform manner. Usually, it occurs in patients with leprosy who relapse after dapsone monotherapy or inadequate antileprotic treatment; it can even arise *de novo*. We present a case of umbilicated presentation in a patient with a case of relapse HL, even after adequate treatment with multibacillary multidrug therapy for 12 months. Although rare, the higher load of lepra bacilli in these cases makes it a matter of concern, and challenging for leprologists to make early diagnosis and treatment.

## Teaser

Relapse and unusual lesions in histoid leprosy.

What is already known about this topic?

- Histoid leprosy (HL) is a highly transmissible form of lepromatous leprosy, presenting as skin-coloured papules, nodules and plaques.
- Usually, it occurs in patients with leprosy who relapse after dapsone monotherapy or inadequate antileprotic treatment; it or can even arise *de novo*.

What does this study add?

- The presence of central umbilication in several lesions in our patient is an atypical feature as only a few cases of HL with umbilicated lesions have been reported in the literature.
- Relapse in our patient could have been caused by persistence or reinfection with *Mycobacterium leprae*, even after adequate and complete treatment with 12 months of World Health Organization multibacillary multidrug therapy.

Histoid leprosy (HL) is a rare and highly infectious variant of lepromatous leprosy, first described by Wade in 1960. It is characterized by skin-coloured papules, subcutaneous nodules and plaques on apparently healthy skin, with distinct histopathology and bacterial morphology.^1^ The term ‘histoid’ was coined after the classic histopathological appearance of histiocytes that form interlacing bands and whorls. HL was initially reported to be due to failure of long-term dapsone monotherapy or irregular or inadequate therapy; however, it now thought that HL can also develop *de novo*. The incidence of HL has been reported to vary from 2.8% to 3.6% in all patients with leprosy.^2^ The diagnosis is made by clinical presentation, high bacterial index in slit skin smear, histopathological examination and lepromin test.^3^

## Case report

**Figure 1:**
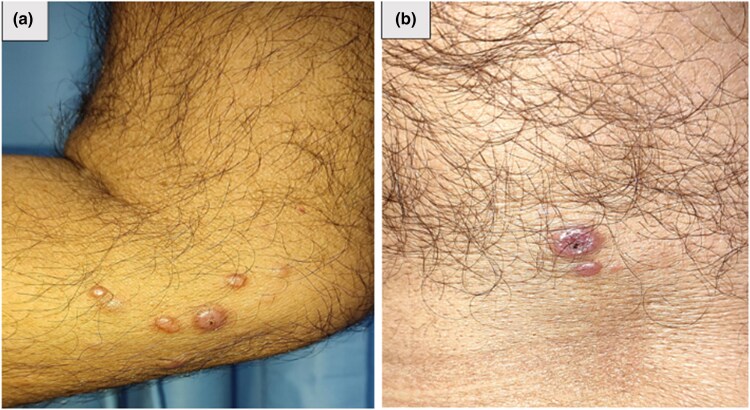
(a) Histoid leprosy: few skin-coloured, shiny, papules over the patient’s right elbow. (b) Central umbilication in a histoid leprosy lesion on the patient’s back.

A 62-year-old man presented with asymptomatic multiple papular skin-coloured lesions of 3 months’ duration. The lesion first appeared on the shin of his right leg and progressed in number to involve his left leg, bilateral thighs, bilateral arm, posterior trunk, lower abdomen, dorsum of the right hand and his right ear. The skin lesions were well-­defined, discrete, firm, shiny, dome-shaped papules and nodules of 5 mm to 1 cm in size with central umbilications (Figure 1). There was no history of leprosy among family members and the patient could not recall social contacts.

The patient had received with standard multibacillary multidrug treatment (MB-MDT) for 12 months for borderline lepromatous leprosy at a regional leprosy centre 10 years previously. Lesions improved clinically following the treatment and the patient was released from treatment after completing 12 blister packs.

Peripheral nerve examination revealed enlarged and nontender bilateral ulnar nerve and left common peroneal nerve. On sensory examination, there was loss of sensation to temperature and fine touch over the ulnar aspect of bilateral hand and left foot up to proximal one-third of the anterolateral aspect of leg. On motor examination, there was wasting of hypothenar eminence of bilateral palms. Mucosal and other systemic examinations were normal.

**Figure 2:**
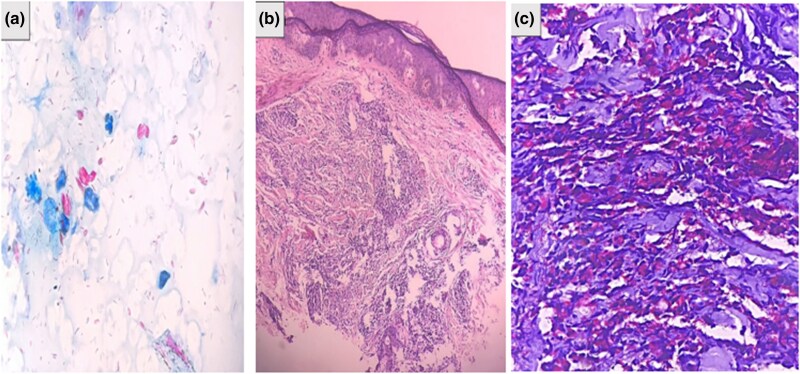
(a) Slit skin smear (Ziehl Nelson stain, ×40): numerous *Mycobacterium leprae* bacilli (pink). (b) Haematoxylin and eosin staining (×10) for tissue section showing spindle-shaped histiocytes and Grenz zone. (c) Fite-Faraco staining (×40) for tissue section showing *M*. *lepre* bacilli (pink).

Slit skin smear from bilateral eyebrows, earlobes and lesions showed a bacterial index of 6+ at each site (Figure 2a). An excisional biopsy of a papule on the right forearm showed spindle-shaped histiocytes in a storiform pattern admixed with foamy histiocytes and Grenz zone. Fite-Faraco stain revealed numerous acid-fast bacilli with a bacterial index of 6+ (Figure 2b, c). The patient met the clinical, bacteriological and histopathological criteria for relapse. Thus, a diagnosis of HL following the relapse was made and he was counselled about the disease prognosis, complications and need for regular follow-up. The patient is responding to the treatment, with no adverse effects recorded up to the 3-month follow-up.

We sent the patient to a national leprosy centre for mouse foot pad inoculation for a drug resistance study; the findings are awaited. The patient was restarted on World Health Organization (WHO) MB-MDT for 24 months and is under regular follow-up.

## Discussion

Leprosy is a chronic granulomatous disease caused by *Mycobacterium leprae*; it affects the cooler parts of the body.^4^ The prevalence rate of leprosy in the community where the patient resides is reported to be 0.38 per 100 000 population.^4^ Nineteen cases of relapse were reported in 2019/2020, which was decreased vs. 2018/2019, as confirmed by Anandaban Leprosy Hospital. However, the overall trend in cases of relapse has increased from 2010/2011 to 2019/2020.^5^

HL is a rare variant of multibacillary leprosy with distinct clinical, histological and immunological features.^2^ HL was originally described in patients who relapsed who took irregular or inadequate antileprotic medications or dapsone monotherapy leading to multiplication of drug-resistant *M. leprae*. However, this explanation is controversial as there have been many case reports of *de novo* HL.^6^ HL presents as well-defined skin-coloured or erythematous shiny papules, plaques and nodules arising from normal skin, most commonly on the back, buttock, extremities and face.^7^ The presence of central umbilication in several lesions in our patient is an atypical feature as only a few cases of HL with umbilicated lesions have been reported in the literature. The pathogenesis involves enhanced local cell-mediated immunity and transepidermal elimination of *M. leprae*.^6^

Histopathological findings include epidermal atrophy, a dermis with a Grenz zone and well-defined collections of fusiform histoid cells in a storiform pattern. Fite-Faraco staining reveals acid-fast bacilli.^7^ Our patient also showed similar histopathological findings. Differential diagnosis of HL includes molluscum contagiosum, dermatofibroma, ­xanthoma, reticulohistiocytosis and cutaneous metastasis.^8^

Relapse in our patients, even after completing 12 months of WHO MB-MDT, could have been caused by persistence or reinfection with *M. leprae*. Persisters are the bacilli that adapt to adverse conditions in their environment by reducing their metabolism to a minimum and are able to survive in their host despite adequate chemotherapy. As Nepal is in the endemic zone for leprosy, reinfection is more common in our part of the world.

Chemotherapy for HL is similar to that for multibacillary leprosy: rifampicin 600 mg once a month supervised, clofazimine 300 mg once a month supervised, dapsone 100 mg daily self-administered and clofazimine 50 mg daily self-administered. As the bacillary load is very high in these patients, all three drugs should be given for at least 2 years, preferably until the slit skin smear is negative.^9^ Close contacts of the patient were screened, and his spouse was given single-dose rifampicin prophylaxis as per current WHO recommendations.

Therapeutic agents like levamisole, thalidomide, zinc, selenium and vitamins A, D, E and C have been tried in various combinations and durations as immunomodulating agents in leprosy.^10^ Bacillus Calmette-Guérin (BCG) vaccine along with *Mycobacterium indicus pranii*, Indian Cancer Research Center (ICRC) bacilli and the new LepVax have also shown efficacy against leprosy.^11^ Our patient had already received BCG vaccination years before and was prescribed vitamin and mineral supplements this time. However, other immunomodulators and immunotherapies were not prescribed to the patient due to unavailability.

The patient, who received WHO MB-MDT, relapsed with HL 10 years after completion of treatment. The detection of new cases of HL in this era is a matter of concern. Therefore, it is necessary to diagnose such cases early and provide adequate treatment, as they are the reservoir of infection and impede the eradication of leprosy.

## Data Availability

The data underlying this article will be shared on reasonable request to the corresponding author.
